# Inherited real risk of Alzheimer's disease: bedside diagnosis and primary prevention

**DOI:** 10.3389/fnagi.2013.00013

**Published:** 2013-03-20

**Authors:** Marco Marchionni, Simone Caramel, Sergio Stagnaro

**Affiliations:** ^1^Department of Neurosurgery, University Hospital of North StaffordshireStoke on Trent, UK; ^2^International Society of Quantum Biophysical Semeiotics, Board of Directors, Research CenterLancenigo, Treviso, Italy

## Introduction

Mitochondria dysfunction (Leuner et al., 2012) and blood-brain barrier (BBB) impairment (Deane and Zlokovic, 2007) are novel researches and insights into the pathogenesis of Alzheimer's Disease (AD), which could lead to classification of AD as a neurovascular disease. Furthermore, on the basis of AD there could be an impairment of neurons response to insulin, that explain the increasing of cerebral blood glycaemia, due to the lowering of insulin receptors and/or of their response. One of the Authors gathered interesting data, due to the fact that there is notoriously an association between high serum cholesterol, raised blood pressure and, finally, insulin-resistance, according with Quantum Biophysical Semeiotics (QBS) theory (Stagnaro and Stagnaro-Neri, 2004b).

QBS theory offers an approach “as a whole” of the patho-physiology of inherited mitochondrial neurodegenerative diseases, as well as that of AD in its various forms, characterized by an Inherited Real Risk (IRR) of Brain Disorders (Stagnaro and Caramel, 2011). In the frame of QBS theory, the combination of Clinical Microangiology and “Angiobiopathy” theory (Stagnaro, 2009a), allows to merge the hypothesis of AD as neurological microvascular—based disease and the researches focused on mitochondrial dysfunction according to the inherited genetic causes of this neurodegenerative pathology, i.e., the congenital alteration of mit-DNA in related neuronal cells. QBS is a new discipline in medical field and an extension of the classical medical semeiotics with the support of quantum and complexity theories. It is a scientific trans-disciplinary approach that is based on the “Congenital Acidosic Enzyme-Metabolic Histangiopathy” (CAEMH) (Stagnaro and Stagnaro-Neri, 1987), a unique mitochondrial cytopathy that is present at birth and subject to medical therapy. The presence of intense CAEMH in a well-defined area (i.e., myocardium) is due to gene mutations in both n-DNA and mit-DNA. This is the basis for one or more QBS constitutions (Stagnaro and Stagnaro-Neri, 2004a) which could bring about their respective IRR (Stagnaro and Caramel, 2012, 2013a,b).

The QBS method allows the clinical and pre-clinical diagnosis of the most severe diseases such as the IRR of brain disorders (Stagnaro and Stagnaro-Neri, 2004b; Stagnaro, 2009a; Stagnaro and Caramel, 2011); this is achieved in the easier way through the auscultatory percussion of the stomach (Stagnaro, 1985a,b, 1986). Made with the aid of gastric aspecific reflex, this diagnosis is consistent and dually reflects the informative nature and quality of parameters collected by QBS microcirculatory investigations. The patho-physiology of QBS reflexes is based upon local microvascular conditions. In case of genetic alteration of both DNAs, intense CAEMH, and IRR of Brain Disorders there is a microcirculatory remodeling, especially intense under environmental risk factors, due to vasomotility and vasomotion impairment (e.g., functional imperfection) and structural obstructions, i.e., pathological Endoarteriolar Blocking Devices (EBDs) and Arteriovenous Anastomosis (AVA) (Stagnaro, 2009c). According to QBS, most of these inherited impairments are already present, in a similar form, in micro-vascular neurobiological systems and clinically observable since birth, through ureteral reflexes diagnosis. Briefly, in healthy, from the microcirculatory point of view, during stress test both vasomotility (chaotic-deterministic oscillations of arterioles) and vasomotility (chaotic deterministic fluctuations of nutritional capillaries and post-capillary venules) are maximally activated (Stagnaro and Stagnaro-Neri, 2004b; Stagnaro, 2009a; Stagnaro and Caramel, 2011), particularly in hippocampus, pre- frontal and parietal cerebral regions. On the contrary, in individuals with a family history positive for AD and, of course, in patients in the first stages of AD, under identical conditions a dissociated form of microcirculatory activation appears, characterized by increased vasomotility and decreased vasomotion. The flow- and flux-motion in the cerebral microcirculatory bed appears to be clearly decreased, due to the dangerous phenomenon of the so-called “microcirculatory blood-flow centralization.” Unfortunately, it is generally admitted that AD diagnosis, particularly in initial stages, is very difficult. The test of acute pick of insulin secretion (Stagnaro-Neri and Stagnaro, 1997) proves to be reliable in bed-side recognizing this (and other numerous) disorder, even in its first stage. Although insulin is not necessary in the glucose utilizations of cerebral neurons, surely in both cerebral cortex and hippocampus there is a large amount of insulin receptors (Craft, 1996). In initial stages of the disease a scarce glucose metabolism in cerebral tissue appears: venous glucose level seems to be slightly decreased (Stagnaro, 2009b). The authors, in addition, showed that O_2_ consumption is unchanged, due to the fact that the neurons utilize other “endocellular” substances rather than glucose, probably causing neurons death (Stagnaro and Stagnaro-Neri, 1987, 2004a,b; Stagnaro, 2009a; Stagnaro and Caramel, 2011).

In summary, in the complex difficult understood pathophysiology of AD a fault response of cerebral insulin receptors exists, while the hormone acts likely as a growth factor. From these work hypothesis, in a previous clinical research we observed that, in healthy, the acute pick of insulin secretion (Stagnaro-Neri and Stagnaro, 1997) activates the microcirculation in all biological systems, while in patients at IRR of AD and, naturally, in patients involved by the disease, even in early stage, microcirculatory activation is totally absent. Interestingly, in no other cerebral disorders, including cerebral arteriosclerosis, it has been observed the absence of insulin-receptors response. From the above remarks, our research is consistent with other authors' theory (Koudinov and Koudinov, 2001), according to which cholesterol is implicated in AD, due to the fact that accurate neuronal cholesterol dynamics is critical for the synaptic plasticity and neural degeneration. These data also imply the link between neuronal lipid metabolism and tau and amyloid beta neurochemistry and propose that the classical AD brain lesions are functional consequences of the neuronal cholesterol and possibly phospholipids biological mis-regulation. In addition, insulin-receptors are less responsive to insulin under such circumstances, as shown previous. The central role of QBS Constitutions and of the IRR of brain disorders in aging people disease occurrence is due also especially if Co Q10 deficiency is present. QBS diagnosis of Co Q10 deficiency syndrome, at bed-side, described earlier (Stagnaro-Neri and Stagnaro, 1990), could be very helpful in risk stratification to predict functional decline in older adults. Doctors can clinically recognize, with the aid of a stethoscope, subjects involved by ubidecarenone deficiency, even initial and symptomless, causing damage of tissues due to the increase levels of free radical (Stagnaro-Neri and Stagnaro, 1992). Such a diagnosis, made clinically for the first time, proved to be really efficacious and reliable in avoiding dangerous administration of statins to individuals without clinical symptomatology, even involved by ubidecarenone deficiency, notoriously worsened by anticholesteremic drugs.

## Inherited real risk of alzheimer's disease: bedside diagnosis

QBS is able to make IRR of brain disorders diagnosis in particular through the auscultatory percussion of the Stomach, easier to understand and apply in the daily practice, i.e., revealing if any subject, from the moment of birth, is at risk of brain disorders.

Among the several QBS signs, one of these is the simultaneous brain gastric aspecific reflex (GAR) in case of “intense” digital pressure on brain's trigger points. This reflex is related with the non-local quantum behavior of biological systems (Stagnaro and Caramel, 2011). In health, “intense” digital pressure on brain's trigger points (any point of the cranium), does not provoke simultaneously GAR (the reflex appears just after 16 s due to physiological tissue acidosis), thus there is not IRR of brain disorders (negative Marchionni's sign): this is the physiological state (Stagnaro, 2009a).

If the stomach moves simultaneously, dilating for at least 1 cm or more, then there is IRR of brain disorders or overt brain disease (if the stomach dilates more than 1.5 cm) termed positive Marchionni's sign. If there is a IRR of brain disorders, in order to discover of which kind of cerebral disease an individual is at risk, the doctors must refine the diagnosis making an investigation more focused on the correct localization of the underlying clinical neurological disorder. This is achieved through QBS assessment of the related specific signs. In fact, this is an aspecific sign, but it becomes specific if the microcirculatory remodeling (Stagnaro and Stagnaro-Neri, 1987; Stagnaro, 2009a; Stagnaro and Caramel, 2011) is present in the typical areas of the related brain disorder, i.e., pre-frontal areas and limbic region, typical areas of AD. There are two main QBS tests able to verify if the IRR of brain disorders is related to AD: the test of insulin secretion acute peak (Stagnaro-Neri and Stagnaro, 1997) and the test of microcirculatory activation of the brain.

In order to apply the insulin secretion test, physicians, after assessing the basal value parameters of the above-described reflexes, immediately thereafter stimulates by lasting pinching the skin of VI thoracic dermathomere for 15 s, repeating the evaluation of the identical reflexes: in both health and patients involved by senile dementia, vascular in origin or mixed, the test improves “always” the numerous parameters mentioned above, although in different manner. For instance, in case of “initial” and asymptomatic senile decay, the latency time of the brain GAR increases from 5–6 s (basal value is 6 s) to 6–7 s (basal value is 8 s). On the contrary, in case of AD, starting from the very initial stage without clinical symptomatology, over years or decades, the acute pick of insulin secretion does not improve at all the above-mentioned parameters values of reflexes, which are identical to those of basal assessment.

In case of intense pressure on brain's trigger points related to AD (limbic region, pre-frontal area, parietal and occipital cortex) if the reflex appear simultaneously there is an IRR of brain disorder linked to AD, but only if the intensity of the reflex is more than 1 cm: positive specific QBS sign due to the fact that the remodeling is present in the typical areas of AD.

## Inherited real risk of alzheimer's disease: primary and pre-primary prevention

QBS tools are not only useful for diagnostic purposes, but also for therapeutic advices, because they are able to measure the microcirculatory activity before and after each preventive therapy's treatment, in order to understand the effectiveness of remedies. Some years ago, one of the authors (Stagnaro and Stagnaro-Neri, 2004a) let us an open question: are QBS Constitutions and IRR of degenerative pathologies reversible?

Through a proper prevention treatment termed “type A” or “green” therapy, i.e., modified Mediterranean diet, CoQ10, conjugated-melatonin, carnitine, a genetic reversibility for future generations is possible (Stagnaro and Caramel, 2013c), but this could not be enough for the current generations, especially under environmental negative conditions. The green therapy stimulates the activity of mitochondria by acting on metabolism, peptides' net, but also improving, normalizing mitochondrial and tissue oxygenation, expression of the normal operation of mitochondrial oxidative phosphorylation. Indeed, the mitochondrial functional cytopathy above mentioned (CAEMH) is the conditio sine qua non of more frequent and severe human disease and not. By this way tissue oxygenation and mitochondrial activity are improved, mitochondria are running well, but it remains the genetic alteration of mit-DNA: CAEMH, QBS Constitutions and IRR of diseases are still positive, but the IRR becomes “residual.” This means that a continuative “type A” therapy averts the risk that the disease can emerge, despite the genetic problem is not yet healed.

QBS method allows an efficient pre-primary prevention with recursive effects able to reverse the genetic alteration of mit-DNA and the mitochondrial cytopathy at the base also of neurodegenerative pathologies such as AD. This is possible under a Type B or blue therapy. In particular, we have successfully used a Quantum Therapy (Stagnaro and Caramel, 2013c,d) for the pre-primary prevention of cancer, Type 2 Diabetes Mellitus, osteoporosis, Coronary Artery Disease and Amyotrophic Lateral Sclerosis. “We are not going to regenerate new neurons but we will stop the decline,” McKew hopes. Well, Quantum Therapy central action mechanism consists in remodeling neurological centers, when heritably altered.

## Conclusions

Alzheimer Disease is not reversible by medical treatments, and the current research is concentrated just on genetic and histological clinical tests. QBS can provide for such disorder a biological preventive evaluation, because biological system functional modification parallels gene mutation. Furthermore QBS is able to make a diagnosis of AD not only at the first very initial stages, usually very difficult to do, but even many years before that such disease could appear, allowing so an efficacious primary prevention, specially prescribing proper preventive treatments, already tested to be really efficacious in healing the IRR of other severe degenerative diseases.
